# Helminth Egg Automatic Detector (HEAD): Improvements in development for digital identification and quantification of Helminth eggs and its application online

**DOI:** 10.1016/j.mex.2020.101158

**Published:** 2020-11-29

**Authors:** Blanca Jiménez, Catalina Maya, Gustavo Velásquez, José Antonio Barrios, Mónica Pérez, Angélica Román

**Affiliations:** Instituto de Ingeniería, UNAM, México

**Keywords:** Automatic identification, Environmental samples, Helminth eggs, Sensitivity, Specificity, Object characterization, TensorFlow, AutoML vision

## Abstract

Conventional analytical techniques for evaluating Helminth eggs are based on different steps to concentrate them in a pellet for direct observation and quantification under a light microscope, which can generate under-counts or over-counts and be time consuming. To enhance this process, a new approach via automatic identification was implemented in which various image processing detectors were developed and incorporated into a Helminth Egg Automatic Detector (HEAD) system. This allowed the identification and quantification of pathogenic eggs of global medical importance. More than 2.6 billion people are currently affected and infected, and this results in approximately 80,000 child deaths each year. As a result, since 1980 the World Health Organization (WHO) has implemented guidelines, regulations and criteria for the control of the health risk. After the initial release of the analytical technique, two improvements were developed in the detector: first, a texture verification process that reduced the number of false positive results; and second, the establishment of the optimal thresholds for each species. In addition, the software was made available on a free platform. After performing an internal statistical verification of the system, testing with internationally recognized parasitology laboratories was carried out, Subsequently, the HEAD System is capable of identifying and quantifying different species of Helminth eggs in different environmental samples: wastewater, sludge, biosolids, excreta and soil, with in-service sensitivity and specificity values for the open library for machine learning TensorFlow (TF) model of 96.82% and 97.96% respectively. The current iteration uses AutoML Vision (a computer platform for the automatization of machine learning models, making it easier to train, optimize and export results to cloud applications or devices). It represents a useful and cheap tool that could be utilized by environmental monitoring facilities and laboratories around the world.

• The HEAD Software will significantly reduce the costs associated with the detection and quantification of helminth eggs to a high level of accuracy.
• It represents a tool, not only for microbiologists and researchers, but also for various agencies involved in sanitation, such as environmental regulation agencies, which currently require highly trained technicians.
• The simplicity of the device contributes to the control the contamination of water, soil, and crops, even in poor and isolated communities.

## Highlights

• The HEAD software will significantly reduce the costs associated with the detection and quantification of Helminth eggs to a high level of accuracy.
• It represents a tool, not only for microbiologists and researchers, but also for various agencies involved in sanitation, such as environmental regulation agencies, which currently require highly trained technicians.
• The simplicity of the device contributes to the control the contamination of water, soil, and crops, even in poor and isolated communities

## Graphical abstract

**Figure:**
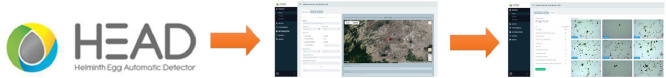

Specifications table

**Table:** 

Subject Area:	Environmental Science
More specific subject area:	Parasitology, automatic detection
Method name:	Helminth Egg Automatic Detector (HEAD): An automatic system for the identification and quantification of different species of Helminth eggs in environmental samples
Name and reference of original method:	*Name and references of the original methods* - *B. Jimenez, C., Maya, Velasquez G., Torner J.F., Arambula F., Barrios J.A. and Velasco M. (2016) Identification and quantification of pathogenic Helminth eggs using a digital image system. Experimental Parasitology, 166: 164-172. https://doi: 10.1016/j.exppara.2016.04.016.* - *B. Jiménez, C. Maya, J.A. Barrios and I. Navarro (2017) Helminths and their Role in Environmental Engineering. Chapter 3. In Luis Rodrigo (Ed). Human Helminthiasis. InTech, 39-62 pp. ISBN 978-953-51-4904-0. DOI: 10.5772/64878.* - *Patent granted in Mexico: Proceso y sistema para la identificación y cuantificación de huevos de helmintos en muestras (MX/a/2013/01 0641) accepted February 14^th^, 2017.* - *Patent USA 9,773,154 B2, accepted September 26th, 2017.* - *Pantent Mexico MX/a/2015/015024. Mexico 28-Oct-2015. Patent MX/a/2015/015024, accepted April 16th 2019*
Resource availability	**Personnel**- Travel and training visits to 11 different countries to demonstrate the Helminth Egg technique and the applications of the HEAD Software, first and second steps- Development of HEAD Software as an online service- Allocation of the development of HEAD Software as an online service- HEAD Software training and online service validation **Equipment and instruments** The equipment and instrument supplies for each of the eleven laboratories selected for the acquisition and submission of images with sufficient quality in terms of illumination, contrast, and sharpness:- A microscope, Carl Zeiss, Primo Star HAL/LED brand, Full Köhler model, right-handed mechanical with camera tube (phototube), 4x, 10x, 40x and 100x oil plan achromatic objectives, 30w Halogen lamp, and eyepieces WF 10X with 20 mm visual field, mechanical stage travel ranges 75 × 30 mm, and Abbe 0.9 to 1.25 condenser - A digital camera, IDS brand, UI-1480LE-C model, color, resolution 2560 × 1920, 6i/s, sensor type CMOS, interface USB 2.0 - A camera adapter, Zeiss P95-C 1/2" 0.5X Model, F/Primo Star - A USB-mini cable - A Micrometric scale - A 20 micron sieve - A Sedgewick Rafter counting chamber - Slides and coverslips - Pipettes - Plastic containers
	**Hardware** Used in training and online service for internal and external validation: - Three desktop computers - One desktop computer to install the service - Two laptops for travel and training visits - Mathworks^Ⓡ^ license **Materials** - Didactic material (posters)- Reagents (zinc sulfate, Helminth egg concentrates)

## Background

Helminths constitute a major public health problem, particularly in developing countries, and represent a health risk associated with poor sanitation. Globally, more than 2.6 billion people are currently affected and infected by Helminths. Helminthiasis (helminthic diseases) are transmitted through eggs, the infective stage of the life cycle, and their risk is linked to their persistence in the environment (several years), their low infectious dose (1 egg), their high resistance to inactivation processes (chlorine, UV, ozone, etc.), and a high oviposition capacity ([11]; Jimenez et al., [8,9]). In order to reduce health risks, since 1980 the World Health Organization (WHO) have developed and implemented guidelines, regulations and criteria for the control of Helminth eggs in wastewater and sludge for reuse and disposal, respectively, in agriculture. Limits restrict concentrations to less than 1 egg per liter (HE/L) of water or 1 egg per gram of dry sludge (HE/gTS) (WHO, [12]). However, traditional methods to ensure compliance involve the final step of identification and quantification through direct observation under the light microscope, which requires trained personnel, and which may sometimes generate errors.

## HEAD software methodology: first version

To address this situation, Jimenez et al. [8] developed a new processing system for automatic identification and quantification of Helminth eggs (Helminth Egg Automatic Detector, HEAD). To develop the system, several steps were followed. These included selecting the species of Helminth eggs to be considered by the system, setting up a digital image reference database for calibration, selecting appropriate Helminth egg properties and designing the associated algorithms to build the system (system development), together with a validation step. The system was developed step by step in Matlab (MathWorks^Ⓡ^), incorporating different image processing tools and pattern recognition algorithms, as described below, as recommended by different authors ([3,^5^,10]; Acvi and Varol, [1]). The system was tested on a personal computer with an Intel^Ⓡ^ Xeon^Ⓡ^ processor and 8 GB of RAM memory.

### Selection of Helminth eggs species

The system was validated for seven species, selected according to their medical importance and worldwide ubiquity. These were *Ascaris lumbricoides* -fertile and infertile eggs-, *Trichuris trichiura, Toxocara canis, Taenia saginata, Hymenolepis nana, Hymenolepis diminuta*, and *Schistosoma mansoni. Hymenolepis nana* was selected due to the difficulty in correctly identifying these species even for trained technicians, while *Schistosoma mansoni* was chosen because it is a widespread genus relevant to the public health field, especially in Africa and South America (Jimenez et al., [8]).

### Helminth egg image acquisition

Images were taken using a Carl Zeiss AxioLab A1 optical microscope and an Imaging Development Systems UI-1480LE-C-HQ USB2 color camera. To collect homogeneous images, all photographs were acquired using 2560 × 1920 pixel resolution without compression. The images included different stages of egg development, including larval and non-larval eggs, as well as texture and morphological variations within species that may be visually differentiated (e.g. size, number of cells or location of the nucleus, and type of membrane: mamillated or non-mamillated). The species identification at this step was performed by experienced staff in laboratories of the Treatment and Reuse Group (Institute of Engineering, UNAM).

### Training algorithms

Once an object in the sample was detected, it was classified according to its shape (area, perimeter, and eccentricity) and texture properties (entropy, mean gray level, contrast, correlation, and homogeneity). After training the system using the database images, the system was capable of identifying any object in the image by using a nearest neighbor classifier with the Mahalanobis distance metric [14]. Subsequently, two modifications were made to improve the system. Firstly, the median filter was replaced with an Anisotropic diffusion filter [10], in order to increase the definition and the detection of the borders of each object, while smoothing the rest of the image. Secondly, a Gaussian derivative function was applied to further improve the detailed definition in the outer shell of the eggs. In this way, the algorithm presented the information of the detected image in a binarized mode. In order to reduce the number of objects processed by the classifier, two morphological filters were applied, the first with a range of compactness index and the second with a range of areas for each object. In this way, the objects were classified into two groups: those identified as Helminth eggs, and all of the remaining objects, such as bubbles, pollen, pine spores, vegetable waste, yeasts, fat, cell debris, bacteria flocs, crystals, etc.; objects that are frequently misidentified and counted as Helminth eggs.

The parameters used to evaluate the proficiency of the system were:

*Sensitivity (Se):* corresponds to the percentage of true positives in relation to the total number of counted eggs:

(1) $$Se=\;\frac{Tp}{\left(Tp+Fn\right)}$$

Where

Tp is the number of true positives.
Fn is the number of false negatives.

“Tp” was regarded as the number of helminth eggs correctly identified and “Fn” as the number of existent helminth eggs that were not correctly identified in the sample.

*Specificity (Sp)* is the percentage of true negatives in relation to all the objects detected excluding actual eggs:

(2) $$Sp=\;\frac{Tn}{\left(Tn+Fp\right)}$$

Where

Tn is the number of true negatives
Fp is the number of false positives

“Tn” was regarded as the number of objects other than Helminth eggs that were correctly identified as “other objects”, and “Fp” as the number of objects other than helminth eggs that were mistakenly identified as eggs.

## HEAD software: second version

The incorporation of different image processing algorithms to a first version of the HEAD software for the digital identification and quantification of Helminth eggs and its online application, generated a second version with the following improvements:

a. An increase from 7 to 11 species of Helminth eggs (Ascaris lumbricoides, fertile and infertile, Toxocara canis, Trichuris trichiura, Taenia saginata and Taenia solium, Hymenolepsis diminuta, Hymenolepis nana and Schistosoma mansoni, and Ancylostoma duodenale, Necator americanus, Fasciola hepatica and Fasciolopsis buski).
b. A greater precision in the efficiency of recognition of the different species (80% to 97%).
c. The adaptation to analyze a greater variety of environmental samples and not exclusively relatively clean wastewater, such as sludge, biosolids, excreta and soils.
d. The application of an online service.

### Java: A second software version

The second version of the software, based on an open source multiplatform language, Java, was developed to replicate the results of the software previously developed using Mathworks^Ⓡ^. The advantage of this new version was that it did not require the purchasing of a license since the language is under the GNU General Public License (GPL). This version was tested in four microbiology laboratories in three countries: Brazil (Companhia Ambiental do Estado de São Paulo and Universidad de São Paulo, CETESB), Colombia (Pontificia Universidad Javeriana), and France (Faculté de Pharmacie de Nancy). All laboratories provided positive feedback, agreeing that the software was a useful tool that could be implemented in their facilities to provide Helminth eggs identification and quantification services. However, one issue was identified during the validation phase: the different image scales associated with the microscope setup at each laboratory resulted in a different focal length for the camera sensor. This problem lowered the sensitivity and specificity of the results to unacceptable values. To correct for the difference in scale, two procedures were assessed: a) adjusting all the metrics used for detecting the different species of Helminth eggs (such as size, shape, texture, entropy, etc.), and b) correcting the acquired images to the same scale used for software training. Since changes in size can cause the texture values to change and fall outside the defined thresholds for a correct classification of the different species of Helminth eggs included in the system, a methodology was established to preserve the visual appearance and characteristics, such as shape and texture and with it allow their processing by the system.

### Gaussian pyramid method

To scale the images, it was necessary to use a method capable of reducing the size of the image while preserving the original metrics such as shape, size and texture. This was made possible by using the Gaussian pyramid method, which first smooths the image to later subsample it. The result is a smaller image, but it retains the same properties, as well as the same width-to-height ratio as the original. The Gaussian pyramid method was programmed in a Matlab graphical user interface (GUI) and the scaled images were processed in the HEAD system with the Java version (Table 1).

**Table 1:** Results after scaling the images processed with the Java version of the software.

Parameter	Faculté de Pharmacie de Nancy, France	Pontificia Universidad Javeriana, Bogotá, Colombia	Environmental analyses department of the Companhia Ambiental do Estado de São Paulo, Brazil	Environmental Health Department of the Universidad de São Paulo (USP), Brazil
Sensitivity	0.93	0.87	0.88	0.88
Specificity	0.89	0.83	0.87	0.96

### C# .net: a new coding language

After the international validation, it was decided that the scaling process had to be integrated into the system itself and not used as a separate algorithm. In addition, it was considered that the time to process the image should be reduced. To address both issues Java was used to share the program directly to the final users, making it possible to run it on platforms such as Windows, Mac or Linux. However, due to the advantages conferred in terms of updating, the international scope and the direct data collection by an online service, it was later decided to use C# .net coding language. This allowed more versatile code, the software became easier to use as a server-client protocol, the results could be obtained faster and fewer resources than those for the previous software version were required. To migrate the code, the first step was the creation of mathematical libraries to homologate the mathematical operations and functions of the Java code and C#. The reproduction of the system was considered acceptable when the object segmentation for a specific image and the acquisition of such object's characteristics had a variation of +/-2%.

During the development of the C# version the following improvements were implemented:

• Integration of the image-scaling module and the detection system (HEAD). This allows the direct processing of a complete set of images without requiring additional software to prepare (scale) them prior to the identification and quantification steps.
• Determination of the operative limits for the scaling module. To establish the limits of the scaling process, tests were carried out reducing and then resizing images to their original size to check if their original characteristics were modified. The results showed that although the shape and gray level characteristics did not change, this was not the case for the texture descriptors. These changed significantly if the image was magnified more than 200%, resulting in the software classifying a Helminth egg as another species or as a different object, as can be seen in Table 2.

**Table 2:** Scaling/Texture relation with correct classification.

Reduced image size, percentage from the original image (%)	Scaling factor, percentage from the reduced image (%)	Allows a correct classification?
0.1	1000	NO
0.2	500	NO
0.3	330	NO
0.4	250	NO
0.5	200	YES
0.6	170	YES
0.7	140	YES
0.8	130	YES
0.9	110	YES

To avoid misclassification problems, it is recommended not to use images with a scaling factor greater than 200% of the size of the original image, due to the numerical errors which result when a bidimensional extrapolation is applied.

**Fig. 1:**
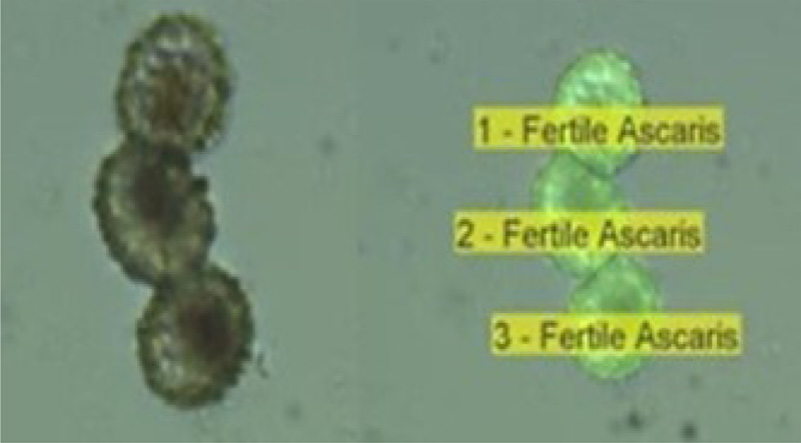
Watershed filter applied in three *Ascaris* eggs adhere.
• Elimination of non-eggs. To reduce the number of false positives, and therefore improve specificity, the Hu first invariant moment was used to eliminate objects that did not meet the thresholds of the software. This is the same methodology that was used for the circularity and diameter-minor axis relation. Another improvement to reduce false positives for fertile *Ascaris* eggs was the implementation of the method proposed by Yang et al. [13], in which the frequency components for the angular trajectory of the probable fertile *Ascaris* egg border were obtained. The objects that are not eggs have more high-frequency components than the average fertile *Ascaris* egg. By establishing a threshold for these values, the software was able to reduce the false positives for this species by 42.85% in CETESB images.
• Reduction of the execution time. In order to optimize the execution time and to minimize the hardware resource demand for the C# version of the software, a reorganization, documentation, and detailed analysis of the algorithms and the main components were performed. Some bottlenecks were detected, such as the Anisotropic diffusion filter, which was solved by applying parallelization and an efficient garbage collector that reduced the execution time by 71% for samples with a low number of objects (water), and by 78% for matrices with a high number of objects (e.g. soils).

• *Segmentation filter.* The presence of transparent debris that adhere to the edges of the Helminth eggs, thereby compromising their proper segmentation, resulted in incorrect sorting and counting in the first version of the software. To combat this, a segmentation filter was installed. This filter is the combination of a Laplacian of Gaussian edge detector with a local threshold (Sauvola), and allowed the correct separation of the eggs from detritus, by a thresholded Laplacian of Gaussian filter. This detects the edges of species such as *Hymenolepis nana*, and was found to be useful for the processing of images obtained from sludge and excreta.
• *Watershed filter.* When, in the same image, two or more eggs adhere, it is necessary to separate them to obtain their shape, gray level and texture characteristics. For this reason, the code includes the Watershed filter that allows separation to be carried out. The name is derived from an analogy with a geological basin, which separates the adjacent basins. The basin algorithm works with the grayscale image since it is a topographic map, with the brightness of each pixel representing its height. Lines that run along the top of the ridges are identified, selecting a threshold to separate connected objects (Fig. 1).

The results for the new implantation of the software in C# improved from those obtained with the Java version as shown in Table 3.

**Table 3:** Comparison of the results obtained with Java and C# versions of the software.

Parameter	Faculté de Pharmacie de Nancy, France		Pontificia Universidad Javeriana, Bogotá, Colombia		Environmental Analyses Department of the Companhia Ambiental do Estado de São Paulo, Brazil		Environmental Health Department of the Universidade de São Paulo (USP), Brazil	
	Java	C#	Java	C#	Java	C#	Java	C#
Sensitivity	0.93	0.91	0.87	0.95	0.88	0.91	0.88	0.90
Specificity	0.89	0.92	0.83	1.00	0.87	0.93	0.96	0.96

### Image scaling algorithm

One of the most important improvements consisted of the development of an image scaling that could be included as part of the main software. The software uses an image of a microscope calibration slide as a reference to adjust the size of the images to match the metrics of the software. For this purpose, an image of such a slide is required for each set of images to be processed by the software. This modification was tested using several images scaled with Matlab and images scaled and analyzed with the new algorithm. The difference between both tests was almost undetectable (i.e. <1%) for size characteristics.

### Mean square contrast (RMS)

An additional improvement was the automatic selection of images whose values for the color and illumination metrics are within the acceptable ranges for their correct classification by the HEAD software. This was obtained through the verification of the illumination uniformity and the mean square contrast (RMS), adding by means of the random collection the values of the background of the image and the calculation of the standard deviation. If the result is outside the predefined threshold, the image will be rejected automatically. The RMS test was determined by the following equation:

(3) $$C_{RMS}=\;\sqrt{\frac{1}{MN}\;\sum_{i=0}^{N-1}\sum_{j=0}^{M-1}{\left(\frac{I_{ij}-\bar{I}}{\bar{I}}\right)}^{2}}$$

Where

$I_{ij}$ is the intensity of the pixel with (i,j) coordinates, M and N are the dimensions of the image and $\bar{I}$ is the average intensity of all pixels values.

## HEAD software vs previous studies

Previous studies using digital images include those carried out by Yang et al. [13] who used a system with an application limited to a few Helminth egg species in clinical samples with a detection rate of 84%; Acvi and Varol [1], and Dogantekin et al. [5] performed the classification/ identification of eggs based on only three characteristics that are not useful to analyze Helminth eggs contained in wastewater samples, since the system is not capable of identifying different species surrounded by debris commonly found in wastewater, sludge or excreta; Holmstrom et al. [7] reported the use a deep learning-based image analysis algorithm to identify three species of helminth eggs (*Ascaris lumbricoides, Trichuris trichiura* and hookworm) in 410 image of fecal samples, where the average sensitivity of the algorithm for the detection was 92%, which is low compared with that of the HEAD system of 97%. Furthermore, the HEAD software represents an automatic system that reduces both the potential for subjective error and the time required for visual identification and quantification under the optical microscope. Its application also confers the advantages of providing uniform criteria for the identification of 11 species of Helminth eggs in different environmental samples (wastewater, sludge, biosolids, excreta, and soils). This reduces the uncertainty of the process, and provides identification in media that so far no other previous study has achieved. The Tensorflow model tested in the present study was trained with 1,188 images which represents a total count of 1773 Helminth eggs examples. A further advantage of the HEAD system is that it can be easily upgraded with more images to reinforce the training.

## HEAD software: allocation on a server for cloud computing service

A quick reference guide was prepared, which describes the software's main characteristics, such as the programming language, the libraries and versions that are used, the framework needed, and the Interface Development Environment (IDE) used in its development. The guide also contains a description of the compatible image properties and formats necessary to use the software. Finally, the it gives step by step directions to run an analysis, from selecting the image data set, scaling it (if needed), and selecting the sample type, how the results may be displayed and where they are stored.

**Fig. 2:**
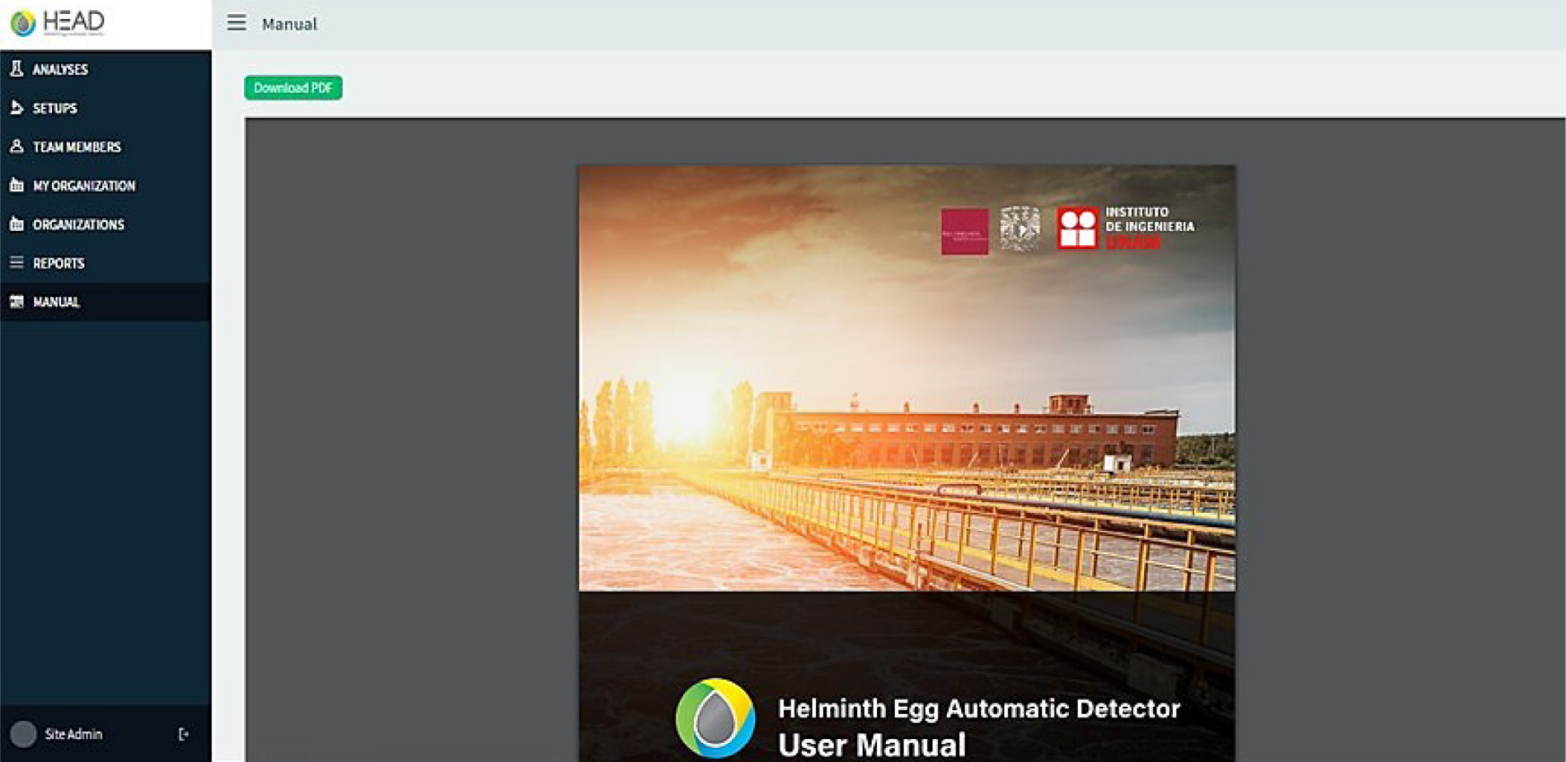
User Manual of HEAD Web application.

• In addition, a group of 450 sample images was prepared for software validation, which included images from different types of samples (wastewater, sludge, excreta, and soil) from different countries (France, Colombia, Brazil, and Mexico). The Image Archive migration has been completed to 90% to the new hosting provider, and reconfigured for high availability, and redundancy.
• Supplementary revisions to integrate The Bill and Melinda Gates Foundation (BMGF) master data scheme and policy to allow data sharing to external systems were incorporated into existing phases. Additional development and implementation of a user management system to allow secured user access to HEAD web application, and administrative functionalities was created. User guidance was additionally built in and provided to end users of the HEAD web application to ensure adoption and compliance with proper usage of the application.
• A "user manual" section was created to provide better advice and an explanation of how the web service works (Fig. 2).

A website has been created that will provide the analysis service with all its benefits, and the registration of the laboratory process, as well as listing the authorized users of it. The system configuration is based on image size, sample type, preservation method and region, and the results of the system are presented by image and by folders of the count of each of the species; likewise, image folders are included to allow remotely processing. The website includes sections such as Analyses, Setups and Team members.

**Fig. 3:**
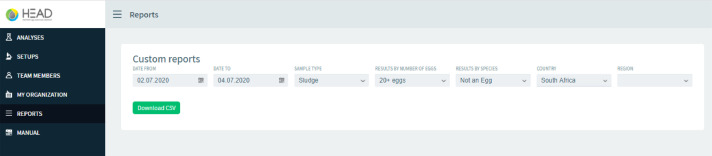
Custom reports section.

**Fig. 4:**
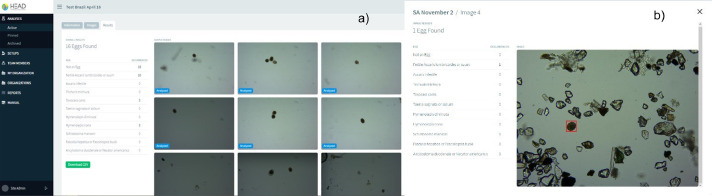
a) General results of the set of images, 4b) Identification in each image.

In the side menu the HEAD website has different options to perform the analysis; the user can navigate to the “Analysis” section, in which a new test can be created. In the “Setups” section, the user can fill system configuration data with their microscope, microscope camera, and calibration image information. In the “Team members” and “My organization” section, the user can edit the organization details. In the “Reports” section an excel file can be created with all the details of the performed test (Fig. 3), and in the final section, “Manual”, the user can find general instructions to work with the online system. In general, the analysis process on the website consists of the identification of the sample, the subsequent selection of an image with a stage micrometric to adjust the scale pixel size and finally the uploading of the Helminth egg images. To create a new analysis, the user fills out all left-hand side fields, which include data such as the date and sample time, type, volume, etc. Once all the images to be analyzed are uploaded, the “Results” tag is available to display the Helminth egg analysis. Fig. 4a shows the general results of a complete set of analyzed images, and the user is able to see the eggs marked in each image (Fig. 4b).

## HEAD software: training and web internal service validation

In order to establish the minimum resolution in which the software maintains acceptable sensitivity and specificity values, training has been reinforced by increasing the amount and variety of images in the database. For this purpose, applications from international laboratories were requested, in particular those that currently perform (or intend to perform) Helminth egg analyses in environmental samples. Microscopes and digital cameras were donated in order to receive images for validation. Once the institutes/laboratories receive the equipment, they are committed to send 50 images monthly from environmental samples containing Helminth eggs over the course of one year. This expanded image database will allow identification of the characteristics prone to more variations between laboratories, even when sharing the same hardware.

In addition, the TensorFlow (open source platform for machine learning) object detection model has been implemented. This replaced the hardcoded training data from the old code version and allows the automatization of the HEAD training process, while also providing a rapid deployment of the cloud computing service. The new model makes the training process easier by only needing a bounding box marker for every egg presented in the image set, instead of manually creating a table with all the characteristics and calculated thresholds values inserted individually into the code [6]. The Tensorflow model generates only one threshold that expresses the similarity between the classified object and the training data set [2,4].

The model was trained on AutoML Vision to detect the presence and location of Helminth eggs. The latest version considers a total number of 1778 egg examples. The image data set was divided into 3 groups: Train (to obtain the egg metrics data to create the object detection model), Validation (images to evaluate the results given during the training), and Test (to provide the general performance of the generated model in an independent group of images). The groups were allocated percentages of 80%, 10%, and 10%, respectively, of the total number of images. The training process needs the first two groups to obtain the egg characteristics and the last one is used to provide the performance of the generated model. Table 4 shows the latest training numbers by Helminth egg species.

**Table 4:** Contribution of each species of Helminth eggs to the training step.

Helminth egg species	Bounding boxes	Train	Validation	Test
Fertile *Ascaris*	869	706	78	85
Decorticated Fertile *Ascaris*	197	167	14	16
*Fasciola hepatica*	138	112	12	14
*Trichuris trichiura*	124	94	17	13
*Taenia solium or saginata*	122	93	12	17
*Toxocara canis*	102	85	8	9
*Hymenolepis nana*	64	48	8	8
*Schystosoma mansoni*	42	33	3	6
Infertile *Ascaris*	40	32	3	5
*Hymenolepis diminuta*	40	33	5	2
Hookworm	35	26	4	5
Total	1773	1429	164	180

It can be seen that the most common species is *Ascaris* with 60% of the count together with the “Decorticated Fertile *Ascaris*” and “Fertile *Ascaris* classes”. The resulting model was generated from different sets of images. For each training image the label of the corresponding eggs and their position in the image is included. When an image is provided to the model, it outputs a list of the eggs it detects, the location of a bounding box that contains each egg, and a score that indicates the confidence that detection was correct. A confidence score is a helpful tool that substitutes all the threshold values for each image egg characteristic, such as area, perimeter, mean gray level, entropy amongst others. This makes it easier to tune the model's sensitivity and specificity results. The score for each detected object is a number between 0 and 1 that indicates confidence that the object was detected. Values close to 1 indicate greater confidence in the classification of eggs. To avoid false positives, a classified object must accomplish a minimum score value to be considered an egg of certain species (confidence threshold). Table 5 shows the confidence threshold value for each species along with its sensitivity and specificity in the test set.

**Table 5:** Confidence threshold and its relation with sensitivity and specificity for each species in a test set.

Helminth egg species	Confidence Threshold Value	True positive	True negatives	False positives	False negatives	Sensitivity (%)	Specificity (%)
Fertile *Ascaris*	0.40	96	316	1	4	96	99
DFertile *Ascaris*	0.36	18	316	5	3	86	98
Infertile *Ascaris*	0.50	5	282	0	0	100	100
*Toxocara canis*	0.50	10	421	0	0	100	100
*Trichuris trichiura*	0.50	11	4938	1	2	85	99
*Hymenolepis diminuta*	0.50	4	359	0	0	100	100
*Hymenolepis nana*	0.50	9	391	0	0	100	100
*Taenia solium or saginata*	0.50	17	357	3	0	100	99
Hookworm	0.28	5	359	3	0	100	99
*Schystosoma mansoni*	0.50	6	592	1	0	100	99
*Fasciola hepatica*	0.50	14	357	0	0	100	100
Total		195	8,688	14	9	96	99

For most of the different species, the confidence threshold value was 0.5 (meaning a 50% probability that the detection is valid). This means that the system will ignore the image objects with confidence scores that are below 0.5. For the cases of Hookworm, Decorticated *Ascaris (*DFertile *Ascaris),* and Fertile *Ascaris* cut-off values of 0.28, 0.36 and 0.4, respectively, were applied.

The cut-off value is decided based on the idea of reducing false negatives (genuine objects that are missed because their confidence was low); however, this may increase the number of false positives (objects that are wrongly identified, or areas of the image that are erroneously identified as objects when they are not). For the validation of the service, preliminary tests of the HEAD online service have been carried out, from the microscopic digital image uploading, to downloading the results in Excel compatible format.

## HEAD software: web international service validation

The online web application was preliminarily tested in all its steps: user register, load images, prequalification test, image processing and downloading the results in Excel compatible format.

**Fig. 5:**
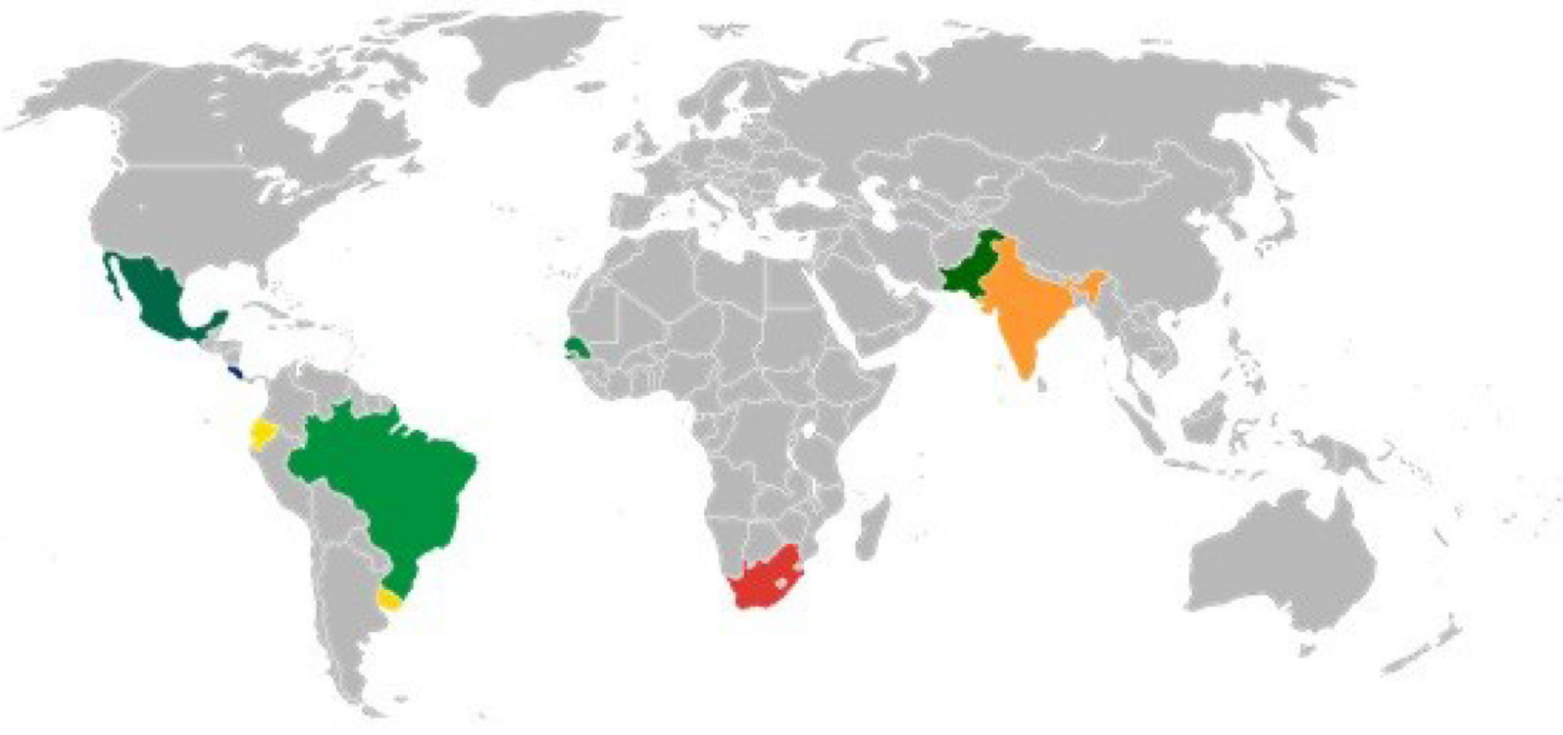
Worldwide laboratories from 9 different countries: Mexico, Costa Rica, Ecuador, Brazil, Uruguay, Senegal, South Africa, Pakistan, and India.
• Shipping of microscopy equipment: The microscopy equipment was acquired by the UNAM (microscopes and digital cameras), and all documentation and paperwork for the exportation needed to be provided to the laboratories was managed and sent to the different countries. The institutes or organizations that participated in this training and validation process and the shipment of the equipment was as follows: the three first microscopy equipment sets were sent to the Instituto Tecnológico de Orizaba in Mexico, the Birla Institute in India and the ISAE laboratory in Brazil, two more shipments were dispatched to NUST University in Pakistan and LATEU lab in Senegal. Four more shipments were dispatched to UdelaR University in Uruguay, UDLA University in Ecuador, the Ministry of Environment in Uruguay, and KwaZulu-Natal University in South Africa. Finally, the last two shipments were sent to the Instituto Costarricense de Acueductos y Alcantarillados in Costa Rica and Amrita School of Biotechnology in India.

• Selection of the international laboratories: Applications from 15 laboratories were received, and 11 laboratories from 9 different countries were chosen. The selection was carried out based on their geographical location, experience in microbiological techniques (preferably in the analysis of Helminth eggs in environmental samples), and potential contribution to diagnosis and controlling Helminthiasis in developing countries. Fig. 5 shows the location of the laboratories selected in the 9 different countries.

## Collection of the homogeneous images

As a first stage, the international laboratories sent the corresponding images via email and once the HEAD website was operational, they proceeded with their online registration so that they could make use of the online service, uploading the images directly once they were registered on the WEB service: 1) Brazil (ISAE), 2) Costa Rica (ICA), 3) Ecuador (UDLA), 4) India (Birla), 5) Mexico (ITO), 6) Pakistan (NUST), 7) Uruguay (MVOTMA), 8) Uruguay (UDLR), 9) Senegal (UCAD), 10) South Africa (UKN), and 11) India (Amrita) To collect homogeneous images, all of them were acquired using:

• 2560 × 1920 pixel resolution without compression.
• Illumination, contrast and sharpness conditions.
• BMP format.
• 10x objective.

Training visits: visits to eight different laboratories that support the project were carried out, focused on the training in the technique for the processing of environmental samples for the personnel that required it and the identification and quantification of Helminth eggs. The activities performed were the following:

• Conferences related to the project in three different languages.
• Support in the Helminth egg technique (training and / or suggestions) in different types of samples.
• Training in the identification of different Helminth eggs species.
• Feedback of experiences with the helminth egg technique.
• Training in image capture:
  - Quality: illumination, contrast and sharpness.
  - Microscope configuration (Köhler illumination).
  - Camera software configuration.
  - Prequalification and evaluation test of the digital images obtained.
  - Registration in the HEAD web system.

As Table 6 shows, the optimal illumination levels for capturing images depends on each country.

**Table 6:** Illumination levels.

Country	Microscope	
	Lens magnification	Illumination
Universidad de las Americas (UDLA) – Ecuador	40x and 10X	1.9
Costarricence de Acueductos y Alcantarillados (ICA) – Costa Rica	10x	1.9
The laboratory of the institute Tecnológico de Orizaba (ITO) – Mexico,	10X	1.9
Institute Superior de Administração e Economia do Mercosul (ISAE) – Brazil	10x	3.0
Laboratory of Wastewater Treatment and Water Pollution (LATEU) – Senegal	10x	2.9

The activities carried out in each laboratory visited are detailed below.

**Fig. 6:**
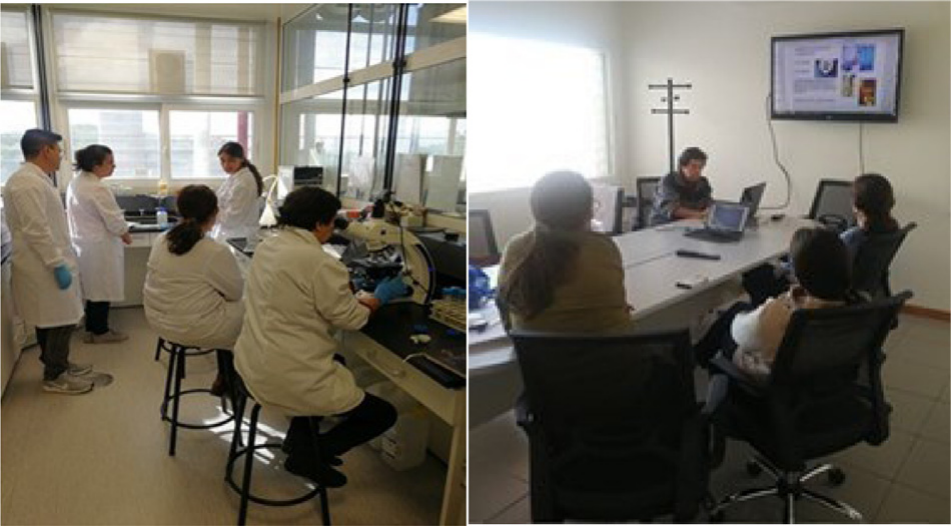
Uruguay MVOTMA.

The laboratory *Ambiental de la Dirección Nacional de Medio Ambiente, Ministerio de Vivienda Ordenamiento Territorial y Medio Ambiente (MVOTMA) – Uruguay* (Fig. 6), was supported with complete training. This Included the development of the technique of concentration, identification and quantification of Helminth eggs, the process of capture and prequalification of images, as well as the definition of optimal lighting conditions.

**Fig. 7:**
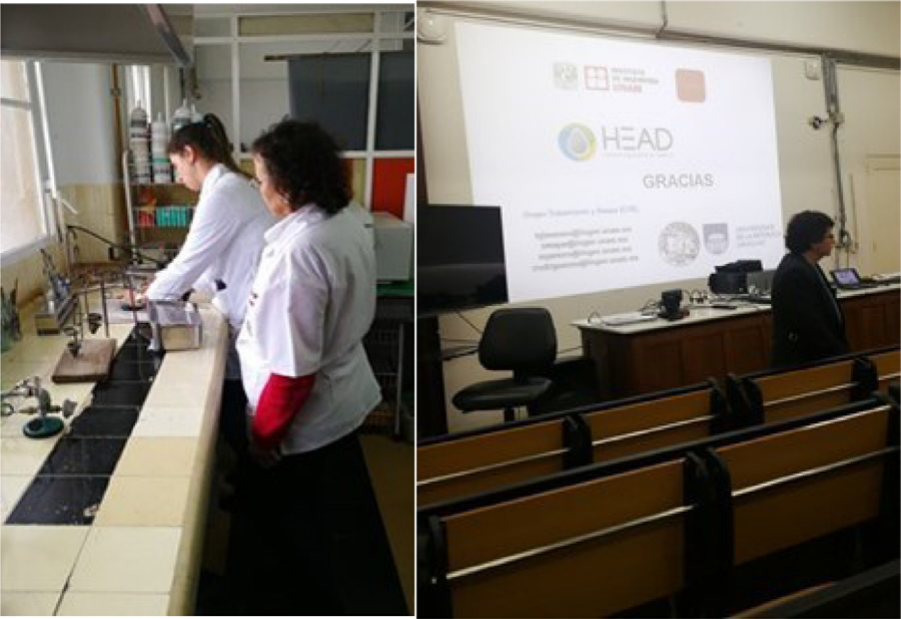
UDLR Laboratory.

For the *Instituto de Higiene (UDLR), Universidad de la Republica-Facultad de Medicina – Uruguay*, the main objective was only to verify the image capture procedure since the participants at this laboratory have extensive experience in the identification and quantification analysis of Helminth eggs (Fig. 7).

In addition to providing support to improve image capture, ideas that could be helpful in improving the technique of identifying Helminth eggs were suggested. For example, the use of a 20 µm sieve enabled cleaner samples to be obtained, i.e. with a lower content of detritus. In addition, a faster reading time was achieved when using a Sedgewick Rafter counting chamber.

**Fig. 8:**
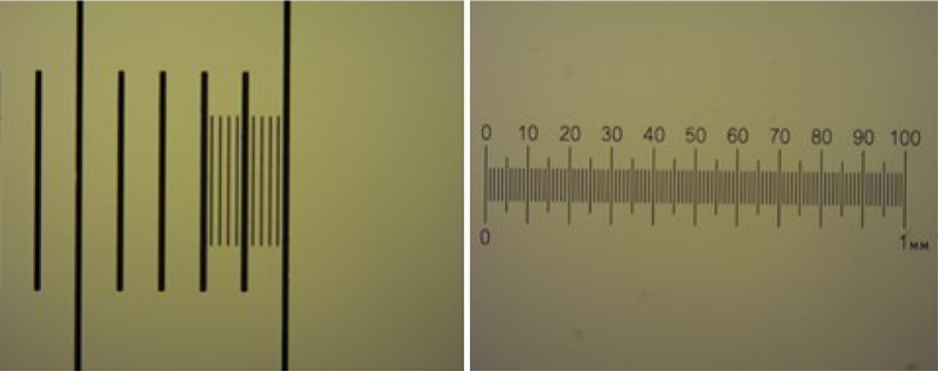
Micrometric scale images, UDLA.

The *Universidad de las Americas (UDLA) – Ecuador,* together with the *Instituto de Higiene - Uruguay,* also has experience in the identification and quantification of Helminth eggs, so it was only necessary to provide support in using the process to verify the quality of the pictures and micrometric scale images (Fig. 8).

**Fig. 9:**
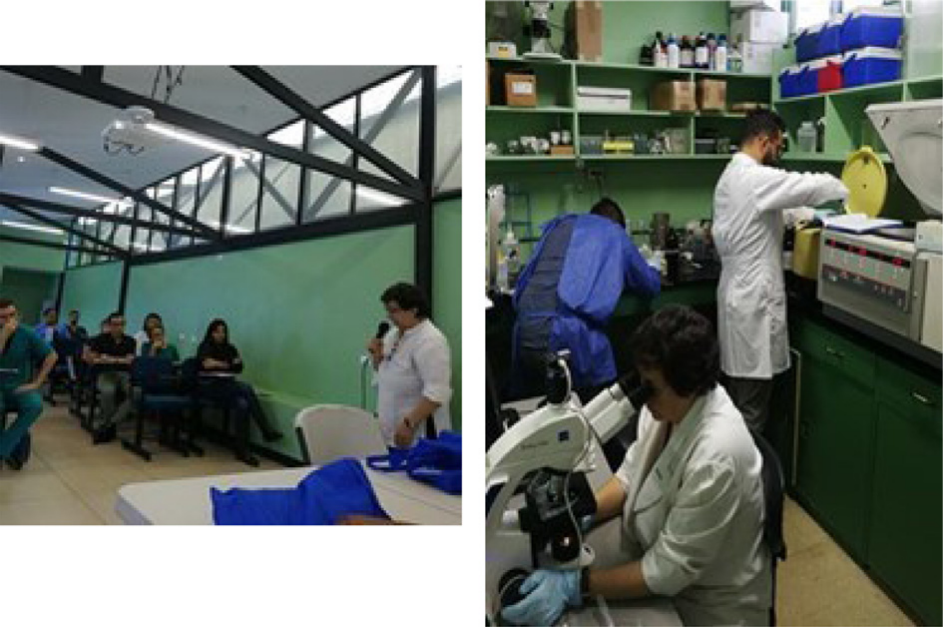
AYA Laboratory.

**Fig. 10:**
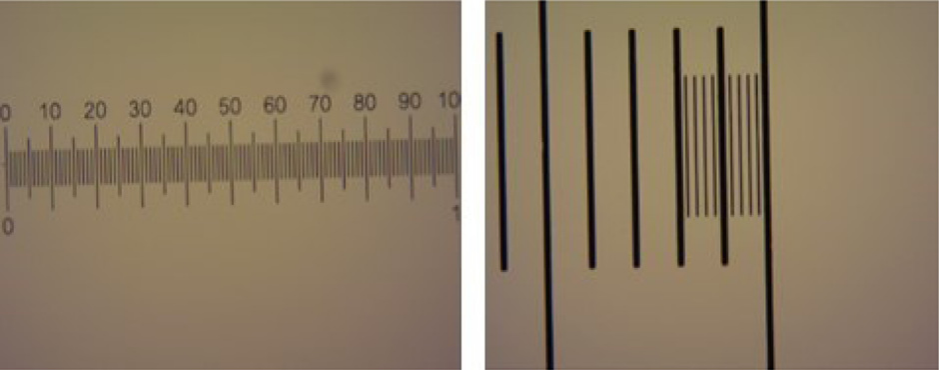
Micrometric scales.

The laboratory of the *Instituto Costarricense de Acueductos y Alcantarillados (ICA) – Costa Rica* (Fig. 9), was also supported by an improvement to the technique used for the detection and quantification of Helminth eggs in biosolids by the implementation of a 20 µm sieve. Additionally, a rapid concentration technique for wastewater samples was developed using the same sieve. The micrometric scale images obtained in this laboratory are shown in Fig. 10.

**Fig. 11:**
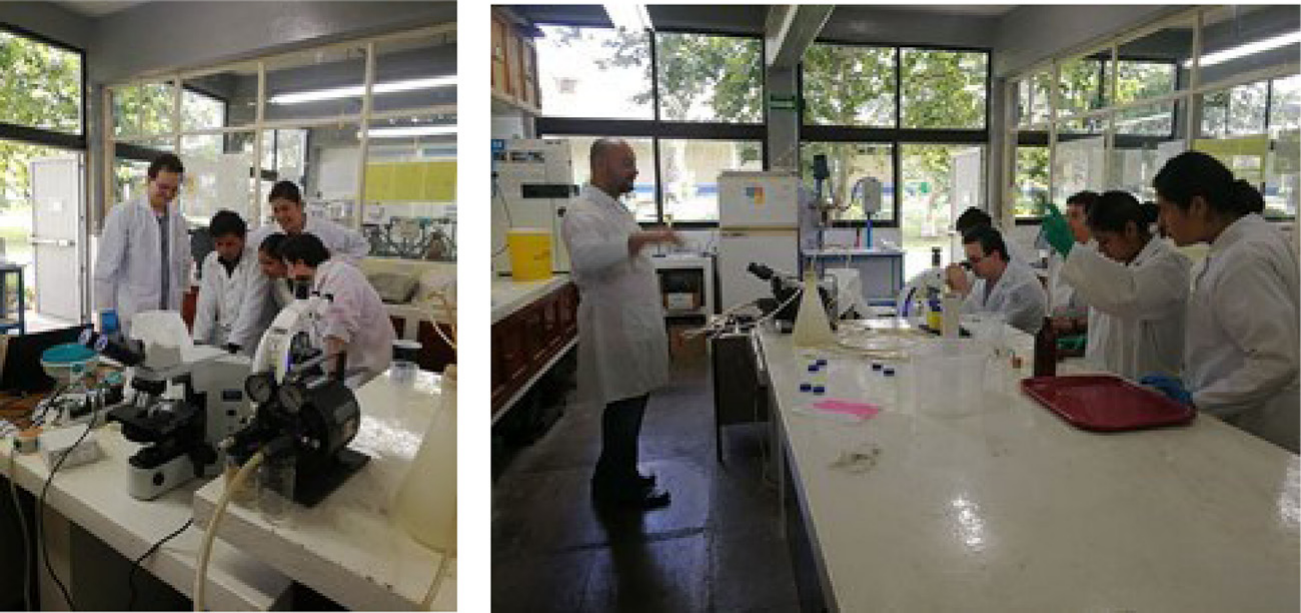
ITO Laboratory.

The laboratory of the Institute *Tecnológico de Orizaba (ITO) – Mexico* (Fig. 11), was also supported in the training of all the techniques used for the detection and quantification of Helminth eggs in wastewater.

**Fig. 12:**
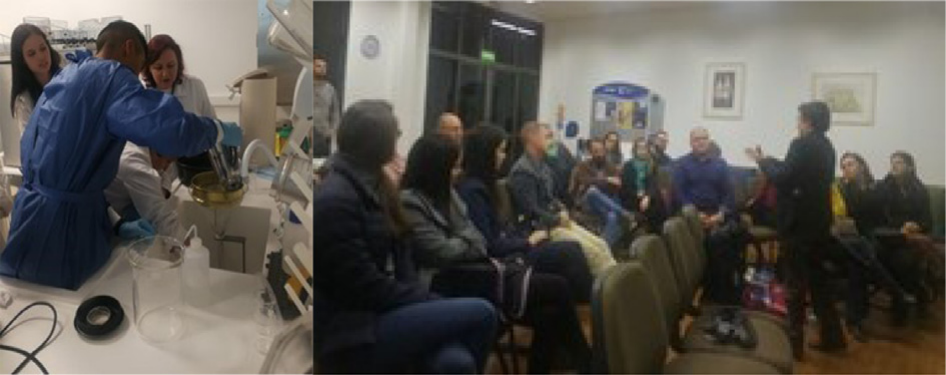
ISAE Laboratory.

The Institute *Superior de Administração e Economia do Mercosul (ISAE) – Brazil* (Fig. 12), was also supported with the development of a rapid technique of concentration, identification and quantification of Helminth eggs in sludge samples, the process of capture and prequalification of images, as well as the definition of optimal lighting conditions. In addition, the laboratory was registered on the HEAD website and the procedure for sending images through the website was explained.

**Fig. 13:**
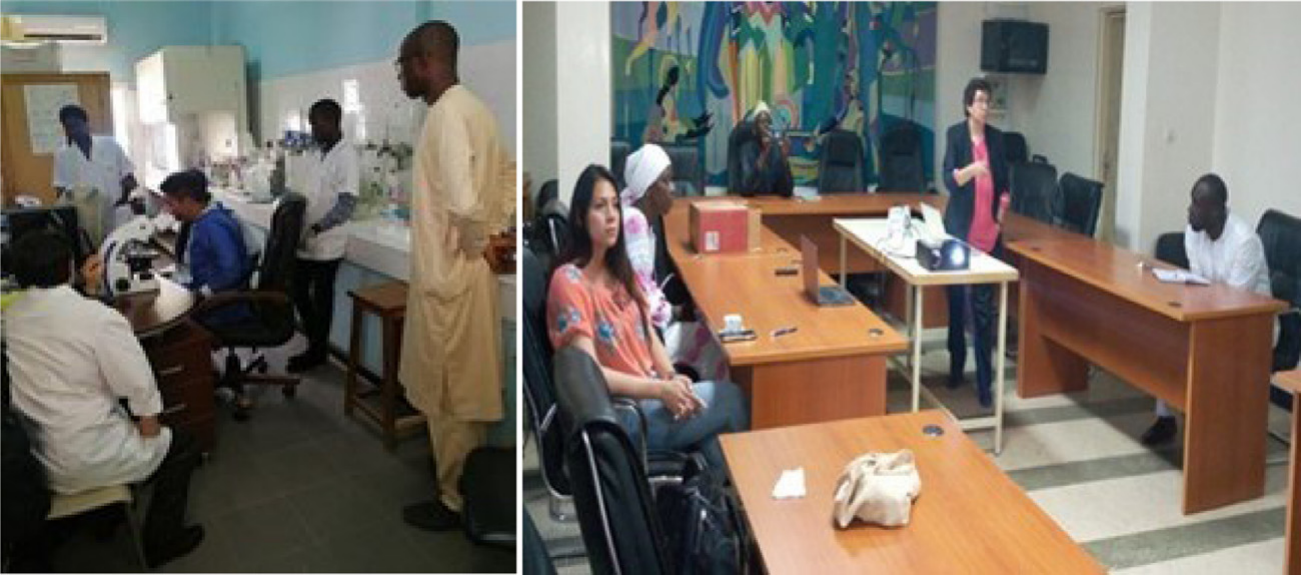
LATEU Laboratory.

The Laboratory of *Wastewater Treatment and Water Pollution (LATEU), University Cheikh Anta Diop* – *Senegal* (Fig. 13) already had experience in the identification and quantification of Helminth eggs. Therefore, the main objective was to verify the image capture procedure, give some recommendations and capture the micrometric scale images. This laboratory was also supported in the registration process and the procedure for sending images through the HEAD website.

**Fig. 14:**
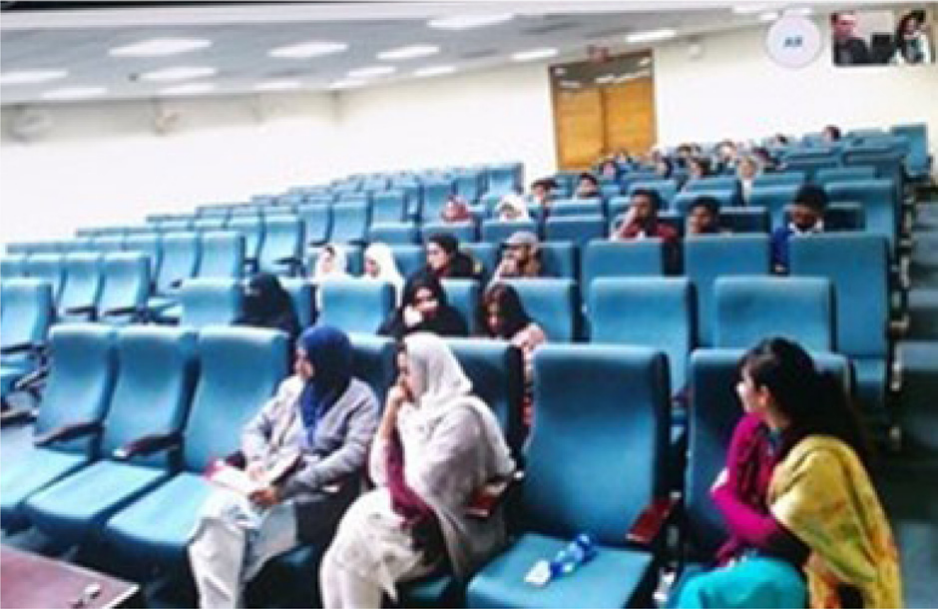
NUST University.

The Department of *Biological Sciences, Birla Institute of Technology & Science – India* and The *Institute of Environmental Sciences* and Engineering (IESE), *National University of Sciences* and *Technology (NUST) - Pakistan* (Fig. 14), was supported through online meetings to clarify doubts regarding the project, image quality and acquisition and use of the HEAD website.

## Conclusions

Enhanced HEAD software is capable of efficiently identifying and quantifying different species of Helminth eggs in various environmental samples, including wastewater, sludge, biosolids, excreta, and soil. It has the advantages of not requiring highly trained personnel and allows consistent and reliable results to be obtained, whose average values in the test image set of sensitivity and specificity are 96.82% and 97.96%, respectively. With the application of the cloud computing service or online client-server it may be used without the need for a data connection application.

The use of this service is expected to reduce identification costs and will provide an option to promptly and reliably detect Helminth eggs within a much wider community. Moreover, the model demonstrated the following advantages:

• It provides uniform criteria for Helminth egg identification, which reduces process uncertainty.
• The flexibility of the image processing tools allows an increase in its identification abilities in terms of water quality of the samples, and the number of species that could be included in the identification database.
• It confers better species classification, due to morphologic and texture characteristics.
• It results in a reduction in the time required for identification and quantification.
• The software will be available as a cloud service.
• It is expected that further steps in the software's development will increase its current capabilities and potential.
• It will provide a valuable tool to improve sanitation conditions and health standards.

The enhanced simplicity provided by the system will be relevant to improve water quality control in many communities in the near future. As a result, the system will provide a valuable tool to improve sanitation conditions and health standards worldwide.

The potential use of this system, not just for Helminth ova analysis but also for other types of pathogenic organisms, demonstrates the software's capability and flexibility to perform similar analyses within the same system, which adds value to its development.
